# Iron overload contributes to general anaesthesia-induced neurotoxicity and cognitive deficits

**DOI:** 10.1186/s12974-020-01777-6

**Published:** 2020-04-11

**Authors:** Jing Wu, Jian-Jun Yang, Yan Cao, Huihui Li, Hongting Zhao, Shuofei Yang, Kuanyu Li

**Affiliations:** 1 Jiangsu Key Laboratory of Molecular Medicine, Medical School of Nanjing University, 22 Hankou Road, Nanjing, 210093 China; 2 Department of Anesthesiology, The first Affiliated Hospital of Zhengzhou University, Zhengzhou, 450000 China; 3 Department of Vascular Surgery, Renji Hospital, School of Medicine, Shanghai Jiaotong University, Shanghai, 200127 China

**Keywords:** Iron, Ferroptosis, General anaesthesia, Neurotoxicity, Cognition

## Abstract

**Background:**

Increasing evidence suggests that multiple or long-time exposure to general anaesthesia (GA) could be detrimental to cognitive development in young subjects and might also contribute to accelerated neurodegeneration in the elderly. Iron is essential for normal neuronal function, and excess iron in the brain is implicated in several neurodegenerative diseases. However, the role of iron in GA-induced neurotoxicity and cognitive deficits remains elusive.

**Methods:**

We used the primary hippocampal neurons and rodents including young rats and aged mice to examine whether GA impacted iron metabolism and whether the impact contributed to neuronal outcomes. In addition, a pharmacological suppression of iron metabolism was performed to explore the molecular mechanism underlying GA-mediated iron overload in the brain.

**Results:**

Our results demonstrated that GA, induced by intravenous ketamine or inhalational sevoflurane, disturbed iron homeostasis and caused iron overload in both in vitro hippocampal neuron culture and in vivo hippocampus. Interestingly, ketamine- or sevoflurane-induced cognitive deficits, very likely, resulted from a novel iron-dependent regulated cell death, ferroptosis. Notably, iron chelator deferiprone attenuated the GA-induced mitochondrial dysfunction, ferroptosis, and further cognitive deficits. Moreover, we found that GA-induced iron overload was activated by NMDAR-RASD1 signalling via DMT1 action in the brain.

**Conclusion:**

We conclude that disturbed iron metabolism may be involved in the pathogenesis of GA-induced neurotoxicity and cognitive deficits. Our study provides new vision for consideration in GA-associated neurological disorders.

## Introduction

Millions of paediatric and elderly patients undergoing surgery require general anaesthesia (GA) each year worldwide. Accumulating evidence is forcing the anaesthesia community to question the safety of GA at the extremes of age [1, 2]. A large body of preclinical as well as some retrospective clinical studies suggest that general anaesthetics (GAs) can have profound and long-lasting effects in young subjects by increasing neuronal death and reducing neurogenesis [3]. On the other hand, elderly patients are recognized to take an increased risk of postoperative cognitive dysfunction (POCD) after GA and surgery [4]. Although GAs have been used clinically for over a century, both their targets of action and the nature of any potentially neurotoxic side effects remain incompletely understood.

GAs can be broadly classified as either volatile inhaled compounds (such as nitrous oxide, sevoflurane, isoflurane, and desflurane) or intravenously administered compounds (such as propofol and ketamine). Two main targets have been extensively described to the GAs: the excitatory *N*-methyl-d-aspartate (NMDA) glutamate receptors and the inhibitory gamma-aminobutyric acid (GABA_A_) receptors. By binding glutamate, NMDA receptors (NMDAR) gate an influx of Ca^2+^ and Na^+^ to cause membrane depolarization, while GABA binds to GABA_A_ receptors, an influx of Cl^−^ results in hyperpolarization [5]. Most GAs, such as ketamine and sevoflurane, activate the inhibitory ion channels and/or inhibit the excitatory ion channels and thereby reduce neuronal excitability and lead to GA [5]. There are other potential targets of anaesthetic relevance including glycine receptors and K^+^ channels [6, 7]. This diversity of potential targets increases the probability of both positive and negative non-anaesthetic effects. NMDA neurotoxicity can be mediated through an iron transport signalling cascade, i.e., NMDAR upregulation might promote iron overload by enhancing DMT1 (divalent metal transporter 1)-mediated iron influx [8], stimulating iron releasing from lysosome [9], thus causing iron-induced cell damage. Therefore, we question whether iron is involved in GA-induced neurotoxicity.

Iron is one of the most abundant trace elements in the human body. Iron plays a critical role in brain development and physiology, neurotransmitter synthesis (including monoamine transmitters and GABA), cytoplasmic protein function, and mitochondrial reactions [10]. Non-transferrin-bound iron (NTBI) in the central nervous system appears to be the main physiologically available iron source, and DMT1 is a very important iron transporter for iron uptake [11, 12]. Excess iron in the brain causes neurotoxicity and significant cognitive impairments, which has been implicated in the pathogenesis of several neurological disorders including hypoxic ischemic brain injury and periventricular white matter injury in neonates [13] and neurodegenerative disorders in elders [10, 14]. Ferroptosis is an iron-dependent regulated cell death process that was discovered recently and is usually accompanied by lipid peroxidation during the cell death. It is closely related to the pathophysiological processes of many diseases [15]. A recent study has shown in vitro that pretreatment with the selective ferroptosis inhibitor, ferrostatin-1, preserved mitochondrial function and mitigated neuronal cell death secondary to isoflurane exposure, suggesting that ferroptosis contributes to isoflurane neurotoxicity [16]. Thus, we propose that disruption of iron homeostasis and iron-dependent ferroptosis contribute GA-induced neurotoxicity.

In this study, we examined the effects of intravenous ketamine and inhalational sevoflurane on brain iron homeostasis, mitochondrial function, and neuronal outcomes in vitro and in vivo. Our hypothesis was confirmed by the findings that GA-induced iron overload and iron chelation by deferiprone (DFP) reversed the neurotoxicity. Moreover, we found that GA-induced neurotoxicity was regulated by NMDAR-RASD1 signalling through DMT1 upregulation.

## Materials and methods

### Animals

Sprague-Dawley rat pups at postnatal day (PND) 6 and 15-month-old male C57BL/6 mice were used in the present study. All experimental procedures and protocols were reviewed and approved by the Animal Investigation Ethics Committee of Nanjing University and were performed in accordance with the Guidelines for the Care and Use of Laboratory Animals from the National Institutes of Health, USA. The animals were housed in a room maintained under standard environmental condition (temperature 22–24 °C, a 12 h light/dark cycle, and 50 ± 10% humidity) with free access to food and water. The rat pups were housed with their mothers till PND 20. At PND 21, the pups were weaned and housed 4–5 per cage in standard condition.

### General anaesthesia

GA was performed based on our previous optimization [17, 18], in which ketamine (75 mg/kg) intraperitoneally or 3% sevoflurane 2 h inhalation daily for three consecutive days can induce neurotoxicity and cognitive impairments. For ketamine GA, rat pups at PND 6 or 15-month-old mice received ketamine (75 mg/kg) intraperitoneally daily for three consecutive days. For sevoflurane GA, animals were put in an anaesthetizing chamber delivered with 3% sevoflurane plus 30% oxygen (O_2_) for 2 h daily for three consecutive days. For control experiments, 30% O_2_ was delivered at the same flow rate. For drug treatment, DFP (75 mg/kg, intraperitoneally, synthesized in China Peptides Co., Ltd., Shanghai, China) or DMT1i (50 mg/kg, orally, MedChemExpress, China) [19, 20] was administered to the animals 1 h before GA daily for three consecutive days.

The composition of the chamber gas was continuously monitored using a DatexTM infrared analyzer (Capnomac, Helsinki, Finland). Animals were kept normothermic throughout the experiment. Animals were kept warm on a plate heated to 37 ± 1 °C and returned to the cage until recovery of the right reflex after GA exposure.

### Rat hippocampal neuronal culture and anaesthetic exposure

Primary neuronal cultures were prepared from embryonic day 16–17 (E16–17) embryos of Sprague-Dawley rats as previously described [21]. Neurons were dissociated and seeded on Poly-Lysine pretreated culture dishes with neurobasal medium, supplemented with B-27, glutamine, antibiotics, and 5% FBS (Invitrogen GIBCO Life Technologies, Carlsbad, CA, USA). After 2 h incubation, primary cultures were maintained in neurobasal medium without FBS in 5% CO_2_ incubator at 37 °C. Half of the culture medium was refreshed every 3 days. After 9 days of culture (DIV 9), the neurons were treated with ketamine (10 μM) in 5% CO_2_ incubator or 3% sevoflurane plus 5% CO_2_ for 6 h at 37 °C, whereas the control group was maintained in same amount of culture medium. For drug treatment, DFP (100 μM) or DMT1i (3 μM) was added to the culture medium 30 min before GA exposure.

### Cell viability assays

The neurons were seeded in 96-well plates for 9 days. Cell Counting Kit-8 (Beyotime Biotechnology) was used to assess cell viability according to the manufacturer’s instructions. Results were expressed as the percentage of reduction of absorbance at 450 nm by calibration with the absorbance of the control cells.

### Lactate dehydrogenase (LDH) assays

LDH is a stable cytoplasmic enzyme present in all types of cells and released into the cell culture medium through damaged plasma membrane. The neuron cells were plated into 12-well plates for 9 days, and LDH release was measured by LDH Assay Kit according to the manufacturer’s instructions (Sigma-Aldrich, USA) following the treatment as needed.

### Mitochondria isolation

Mitochondria from the hippocampus or primary hippocampal neurons were isolated using Qproteome Mitochondria isolation kit (Qiagen, Cat. No. 37612, USA). In brief, tissue or cells were homogenized in ice-chilled Dounce homogenizers using isolation buffer and centrifuged at 1000*g* for 10 min at 4 °C. The supernatant was then centrifuged at 6000*g* for 10 min to separate the mitochondria and cytoplasm. The later mitochondria-enriched pellets were gently resuspended and washed with isolation buffer, then pelleted by centrifugation at 6000*g* for 10 min. The later supernatant was transferred into new EP tubes and centrifuged at 12,000*g* for 10 min at 4 °C. This supernatant was considered as cytosolic fraction. The protein was determined by the Micro BCA protein assay kit (Beyotime Institute of Biotechnology).

### Ferrozine iron assays

Iron content was measured using a colorimetric ferrozine-based assay with some modifications [22]. Briefly, 22 μl concentrated HCl (11.6 mol/L) was added to 100 μl cell lysate (~ 500 μg total protein). The mixed sample was heated at 95 °C for 20 min, then centrifuged at 12,000*g* for 10 min. Supernatant was transferred very gently into fresh tubes. Ascorbate was added to reduce the Fe(III) into Fe(II). After 2 min of incubation at room temperature, ferrozine and saturate ammonium acetate (NH4Ac) were sequentially added to each tube and the absorbance was measured at 570 nm (BioTek EL x 800, Shanghai, China) within 30 min.

### Determination of mitochondrial swelling

To initiate mitochondrial swelling by Ca^2+^ uptake, freshly isolated mitochondria were suspended in mitochondrial suspension buffer [120 mM KCl, 25 mM sucrose, 5 mM KH2PO4, 0.1 mM EGTA, 20 MOPS (pH 7.2)] in the presence of 5 mM malate and 5 mM pyruvate as substrates. Swelling was recorded as the decrease of the density of the mitochondrial matrix at 540 nm with a UV/Vis spectrophotometer after adding 0.5 mM Ca^2+^ into the medium.

### Determination of mitochondrial membrane potential (MMP) levels and ATP contents

Levels of MMP and ATP contents were determined in hippocampal neurons using a 5,5′,6,6′-tetrachloro-1,1′,3,3′ tetraethylbenzimidazolylcarbocyanine iodide (JC-1) mitochondrial membrane potential detection kit (Solarbio Science & Technology Co. Ltd.) and ATP content assay kit (Beyotime Biotech) according to the manufacturer’s instructions.

### Mitochondrial morphology imaging

Mitochondrial morphology was observed under the confocal microscopy following staining with the mitochondria targeting dye MitoTracker Red CMXRos (Beyotime Biotech). After GA treatment for 6 h, the neurons were washed and stained with 20 nM MitoTracker Red CMXRos in neurobasal medium for 30 min at 37°C in dark in a 5% CO_2_ incubator. The cells were then washed with HBSS and immersed in neurobasal medium to prevent cell damage. Images were obtained with confocal microscopy (Fluoview FV 10i, Olympus) and analysed using FV10-ASW 2.1 Viewer software.

### Measurement of cellular ROS, MDA, and GSH levels

Intracellular reactive oxygen species (ROS) levels were estimated using a fluorescence-labelled probe DCFH-DA (Beyotime Biotech). Cellular malondialdehyde (MDA) and glutathione (GSH) levels were measured following the manufacturer’s instructions (Nanjing Jiancheng Bioengineering Institute, Nanjing, China).

### Western blot analysis

Proteins were obtained from the hippocampal tissue or cultured neurons or isolated mitochondria. For western blotting, 35–50 μg protein was added per lane of 12% SDS-PAGE. Primary antibodies were diluted in primary antibody dilution buffer (Beyotime Biotech., Shanghai, China). Antibodies used included the following: anti-GAPDH (Abgent Biotech. Co. Ltd., Suzhou, China), anti-ferritin, superoxide dismutase 2 (SOD2), Mitoferrin1, Drp1, Mfn2, RASD1, NMDAR1, and NMDAR2A (Abcam, Cambridge, MA, USA), anti-TfR1 (Zymed, San Francisco, CA, USA), anti-IRP2 (polyclonal, raised from rabbit), and anti-DMT1 (Alpha Diagnostic International, San Antonio, TX, USA). Detection was performed using peroxidase-conjugated secondary antibodies (Thermo Fisher Scientific, Waltham, UK). Quantification of the density of the western bands was done with programme ImageJ (http://rsb.info.nih.gov/ij/).

### Morris water maze tests

To investigate spatial learning and memory function, we subjected the rats (*n* = 12 for each group) to the Morris water maze (MWM) tests (XR-XM101; Shanghai Xinruan Information Technology Co., Ltd., Shanghai, China) at PND 60. The MWM was a black metal tank (120 cm in diameter, 60 cm in depth) equipped with a platform (10 cm in diameter) 1–2 cm below the surface of the water. The MWM task was performed according to our previous study [18]. Briefly, it consists of two phases, training phase for five consecutive days and probe trial phase on day 6. In the training phase, the rat was allowed to face to the pool wall in four random places (N, S, E, W) in the pool to find the fixed platform. Release positions were randomly predetermined. The trial was terminated once the rat reached the platform. If the rat failed to reach the platform within 60 s, it would be guided to the platform and allowed to stay for 15 s, then the latency was recorded for 60 s. In the probe test, a single-probe trial was conducted with the original platform removed 24 h after the last training session. The rat was released at a random start position and allowed to swim for 60 s in the pool.

### Fear conditioning tests

To measure the abilities of learning and memory, we employed the fear conditioning paradigm (30 cm long × 26 cm wide × 22 cm high, XR-XC404, Shanghai Softmaze Information Technology Co. Ltd.) to the aged mice 24 h after GA. Each mouse was exposed in the conditioning chamber for 3 min of accommodation then one tone-foot-shock pairing (tone, 30 s, 65 dB, 3 kHz; foot-shock, 3 s, 0.75 mA) was delivered. The contextual fear conditioning test was performed 24 h later by placing mice back to the same test chamber for 5 min without any stimulation. Two hours later, each mouse was placed in a novel chamber altered in shape, colour, and smell, and the same tone was presented for 3 min without the foot shock to evaluate tone fear conditioning. Cognitive deficits in the test were assessed by measuring the length of time of “freezing behaviour,” which is defined as a completely immobile posture except for respiratory efforts. Freezing behaviour was automatically recorded by the video tracking system.

### Statistics analysis

Data were presented as mean ± SEM and analysed by the Statistical Product for Social Sciences (SPSS; version 18.0, IL, USA). The difference between the groups was determined by one-way analysis of variance followed by the Bonferroni test. Comparisons for the spatial training sessions of MWM were performed by repeated two-way ANOVA followed by the LSD test. A *p* value < 0.05 was considered statistically significant.

## Results

### GA causes hippocampal iron overload

**Fig. 1:**
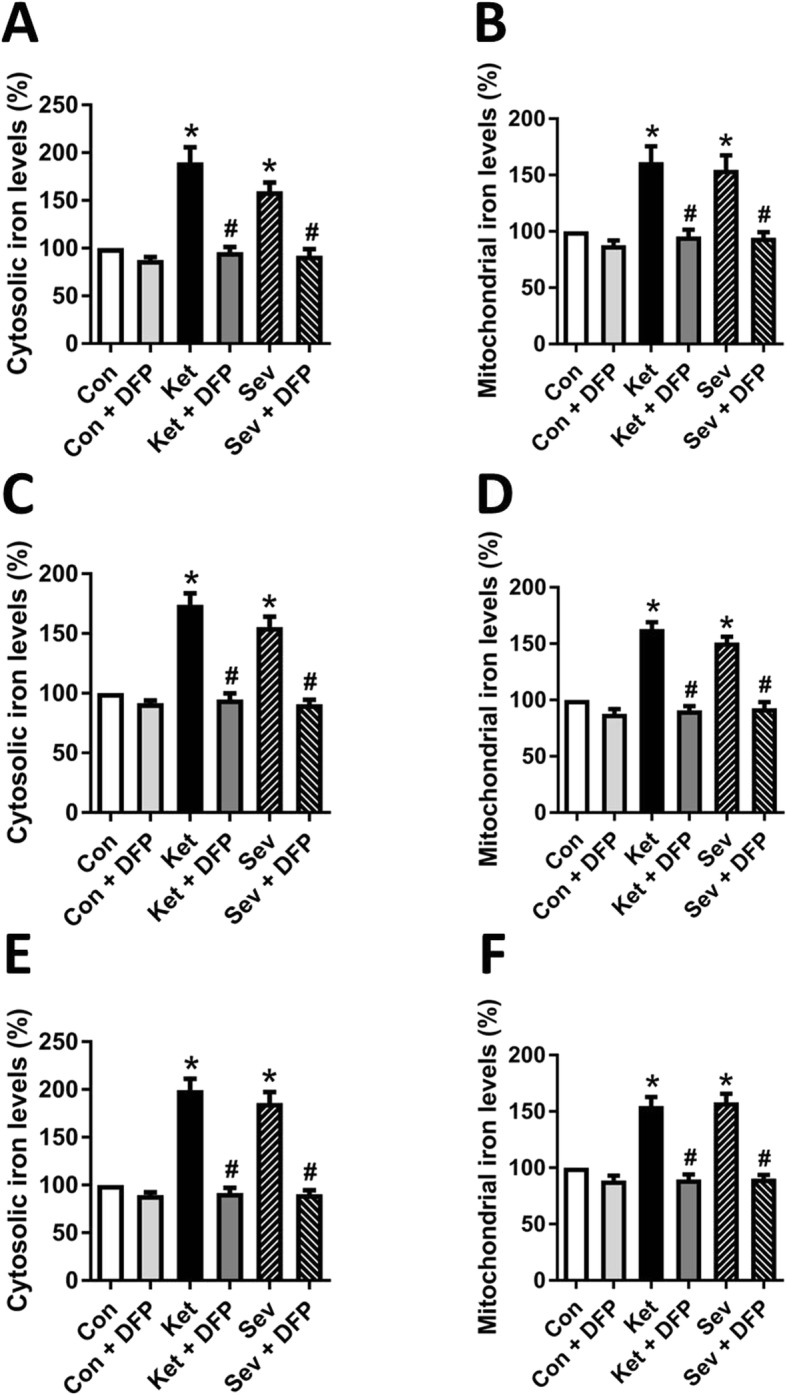
GA induces hippocampal iron overload. **a**, **b** The hippocampal neurons were exposed to ketamine (Ket, 10 μM) or 3% sevoflurane (Sev) for 6 h at day 9 in vitro (DIV 9). The cytosolic iron levels (**a**) and mitochondrial iron levels (**b**) of hippocampal neurons were measured. **c**, **d** The rat pups at postnatal day (PND) 6 received ketamine (75 mg/kg) intraperitoneally or 3% sevoflurane 2 h inhalation daily for three consecutive days. The cytosolic iron levels (**c**) and mitochondrial iron levels (**d**) of rat hippocampus were measured. **e**, **f** The 15-month-old mice received ketamine (75 mg/kg) intraperitoneally or 3% sevoflurane 2 h inhalation daily for three consecutive days. The cytosolic iron levels (**e**) and mitochondrial iron levels (**f**) of the mouse hippocampus were measured. Data are presented as mean ± SEM (*n* = 6); *p < 0.05 compared with Con group; ^#^*p* < 0.05 compared with the GA (Ket or Sev) group

Multiple or long-time exposure to GAs may produce profound and long-lasting effects in extremes of lifetime by reducing neurogenesis and/or accelerating neurodegeneration. Iron deposition in the hippocampus, cortical areas, and basal ganglia has been revealed to impair the spatial memory, aversive memory, and recognition memory [10]. To test our hypothesis that GA would induce iron overload, which could be involved in GA-induced neurotoxicity in the brain, we first measured the iron content in cultured hippocampal neurons and in the hippocampus of developing rats and aged mice after exposure to ketamine or sevoflurane. The results showed that both ketamine and sevoflurane caused cytosolic and mitochondrial iron accumulation in cultured hippocampal neurons (Fig. 1a, b) and in the hippocampus of developing rats (Fig. 1c, d) and aged mice (Fig. 1d, e).

Deferiprone (DFP) is a low-molecular weight iron chelator, which has been clinically used to treat iron overload, particularly for patients with haemosiderosis. DFP can freely cross the blood-brain barrier and bind iron in multiple subcellular and extracellular locations [23, 24]. To see if DFP would reverse the GA-induced overload, we treated the neurons 30 min or animals 1 h before GA exposure. The results showed that the effect of ketamine or sevoflurane on the contents of cytosolic and mitochondrial iron was prevented (Fig. 1). These findings suggested that both intravenous and inhalational GAs could cause iron overload in hippocampal neurons and in the developing and aged brains.

### GA disturbs neuronal iron metabolism

**Fig. 2:**
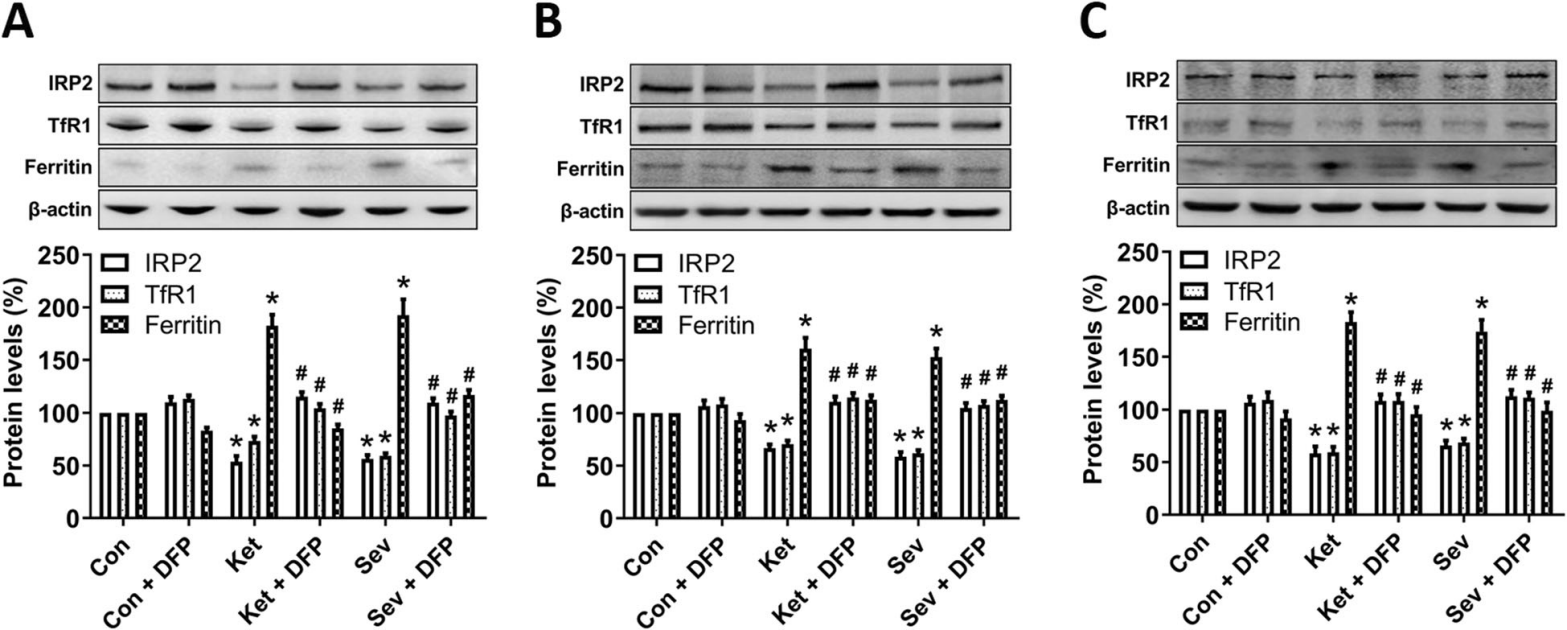
GA disturbs neuronal iron metabolism. Western blot analysis of iron-related proteins including IRP2 (iron regulatory protein 2), TfR1 (an iron uptake protein), and ferritin (an iron storage protein). A representative image set is presented for the cultured hippocampal neurons at DIV 9 (**a**), the hippocampus of rat pups at PND 8 (**b**), and the hippocampus of 15-month-old mice (**c**). The drug treatment is same as in Fig. 1. Data are presented as mean ± SEM (*n* = 6); *p < 0.05 compared with Con group; ^#^*p* < 0.05 compared with the GA (Ket or Sev) group

Cells maintain optimal intracellular iron levels by iron regulatory proteins (IRP1 and IRP2), which can posttranscriptionally regulate the expression of a set of genes such as transferrin receptors 1 (TfR1) for iron uptake and ferritin for iron storage. Therefore, we measured the expression levels of these proteins after ketamine or sevoflurane exposure. Our results showed that both ketamine and sevoflurane reduced the expression of IRP2 and TfR1 and increased the expression of ferritin in the cultured hippocampal neurons (Fig. 2a) and in the hippocampus of developing rats (Fig. 2b) and aged mice (Fig. 2c), respectively. Moreover, depletion of iron with DFP successfully revered the iron status (Fig. 2).

**Fig. 3:**
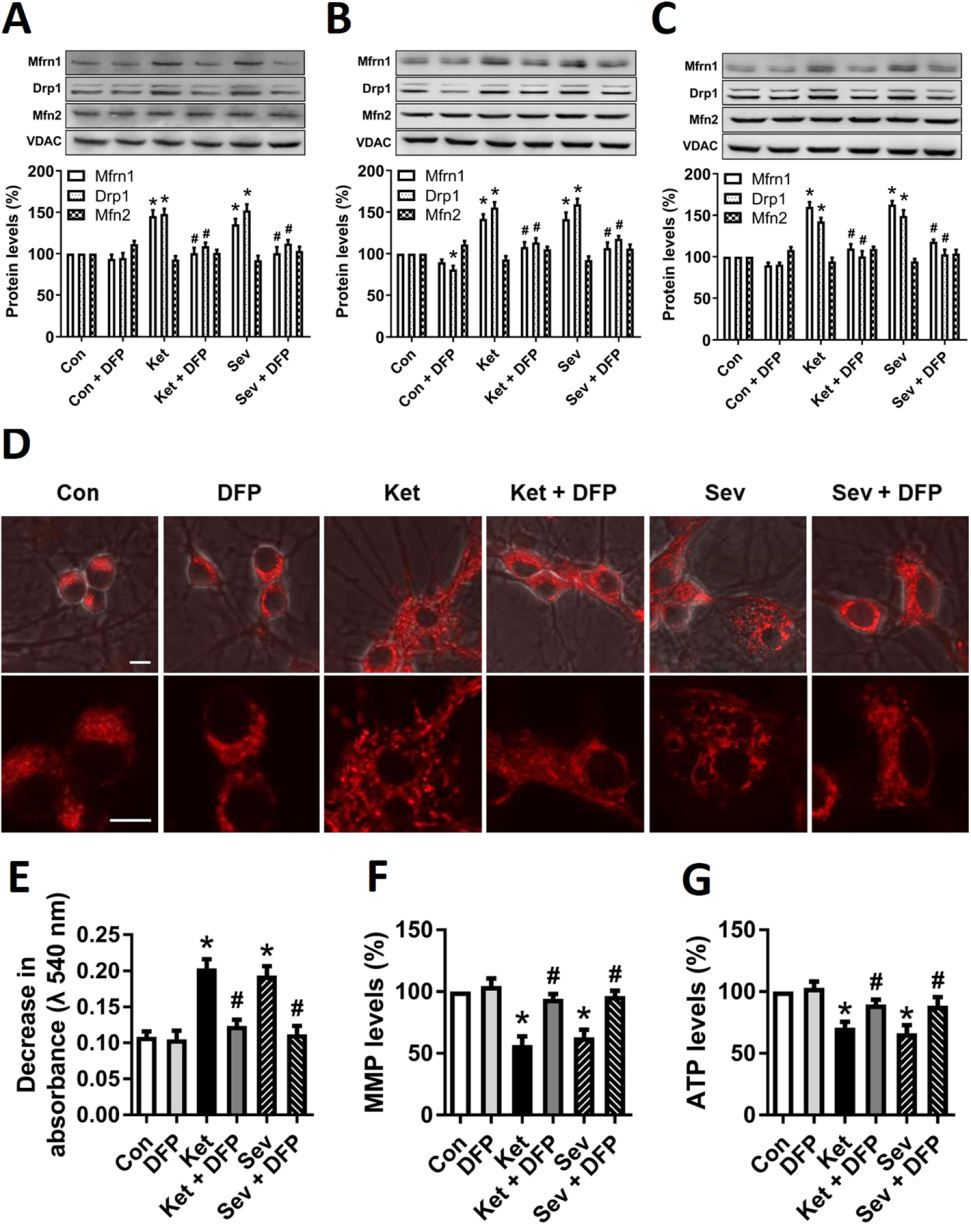
GA causes neuronal mitochondrial dysfunction. **a**–**c** Western blot analysis of mitochondrial iron import protein Mfrn1, mitochondrial fission protein Drp1, and fusion protein Mfn1. A representative image set is presented. Tissues or neurons were lysed and mitochondria were isolated from rat hippocampal neurons (**a**), rat-pup hippocampus (**b**), and aged-mouse hippocampus (**c**). **d** Mitochondrial morphology. After GA treatment for 6 h, the hippocampal neurons were stained with MitoTracker Red at 37 °C for 30 min and observed under the confocal microscopy at × 600 magnification. Scale bar 10 μm. **e** Mitochondrial permeability transition pore (mPTP) opening, determined by mitochondrial swelling assay kit with freshly isolated mitochondria. **f** Mitochondrial membrane potential (MMP) levels. **g** ATP production. Data are presented as mean ± SEM (*n* = 6); *p < 0.05 compared with Con group; ^#^*p* < 0.05 compared with the GA (Ket or Sev) group

Mitochondria play a vital role in cellular iron metabolism. These organelles are the major hubs of iron utilization and the sole site of heme synthesis and the major site for biogenesis of iron-sulfur clusters, which are vital for cell functions [25–27]. The mechanisms of mitochondrial iron transport have not been completely revealed. The best known pathway is inward iron transport mediated by mitoferrin (Mfrn), a protein located in the inner mitochondrial membrane [25, 28]. In this study, we found that the Mfrn1 level was significantly increased in the GA-treated groups when compared to the control group in the cultured hippocampal neurons (Fig. 3a) and in the hippocampus of developing rats (Fig. 3b) and aged mice (Fig. 3c). Taken together, it is demonstrated that both ketamine and sevoflurane intensify the import of iron into cytoplasm and mitochondria, resulting in the accumulation of iron in the cells or tissues.

### GA-induced iron overload causes mitochondrial dysfunction

Neurons are very sensitive to iron overload, of which the toxicity may lead to neuronal mitochondrial dysfunction [25–27]. We have previously demonstrated that mitochondrial dysfunction is involved in the earliest pathogenesis of GA-induced cognitive impairments in developing or aged rodent brain [17, 29]. Whether the GA-induced iron overload elicits mitochondrial dysfunction is not understood. To address this question, we investigated the iron involvement in between GA and mitochondrial dysfunction.

Mitochondria are highly dynamic organelles that continuously divide and fuse through the processes of fission and fusion. First, we measured the protein levels of Drp1 and Mfn2, which are fission and fusion-related proteins, respectively. The result showed that the levels of Drp1 elevated and no significant variation of Mfn2 levels was observed after ketamine or sevoflurane exposure, whereas iron chelation by DFP reversed the expression of Drp1 (Fig. 3a–c), suggesting that iron overload induced by GA exposure led to the mitochondrial fragmentation. Then, we examined the mitochondrial morphology after incubation of the hippocampal neurons with mito-tracker by the confocal microscopy following GA exposure. Consistent with the changes of mitochondrial dynamic proteins, mitochondria became fragmented and condensed after 6 h of ketamine or sevoflurane treatment (Fig. 3d). Finally, we evaluated the mitochondrial function by determining the levels of mitochondrial permeability transition pore (mPTP) opening, MMP, and ATP production, three important parameters of mitochondrial function-related indexes. The results showed that the opening of mPTP (Fig. 3e) was enhanced, and MMP (Fig. 3f) and ATP production (Fig. 3g) were reduced after GA exposure. However, all the GA-induced effects were prevented by pretreatment with DFP as shown in Fig. 3.

Collectively, these data support that mitochondrial dynamics and function are impaired by ketamine or sevoflurane exposure and iron chelation may ameliorate the injury.

### GA triggers iron-mediated ferroptosis

Ferroptosis is an important cell death pathway for a number of diseases, including cancer and neurological disorders [15]. It can be prevented by iron chelators such as DFP, deferoxamine (DFO), ciclopirox (CPX), and 2,2-bipyridyl (2,2-BP) [30, 31]. The selective ferroptosis inhibitor, ferrostatin-1, is able to mitigate neonatal isoflurane-induced neuronal death [16]. Here, we hypothesize that ferroptosis may play an important role in both ketamine- and sevoflurane-induced neurotoxicity.

**Fig. 4:**
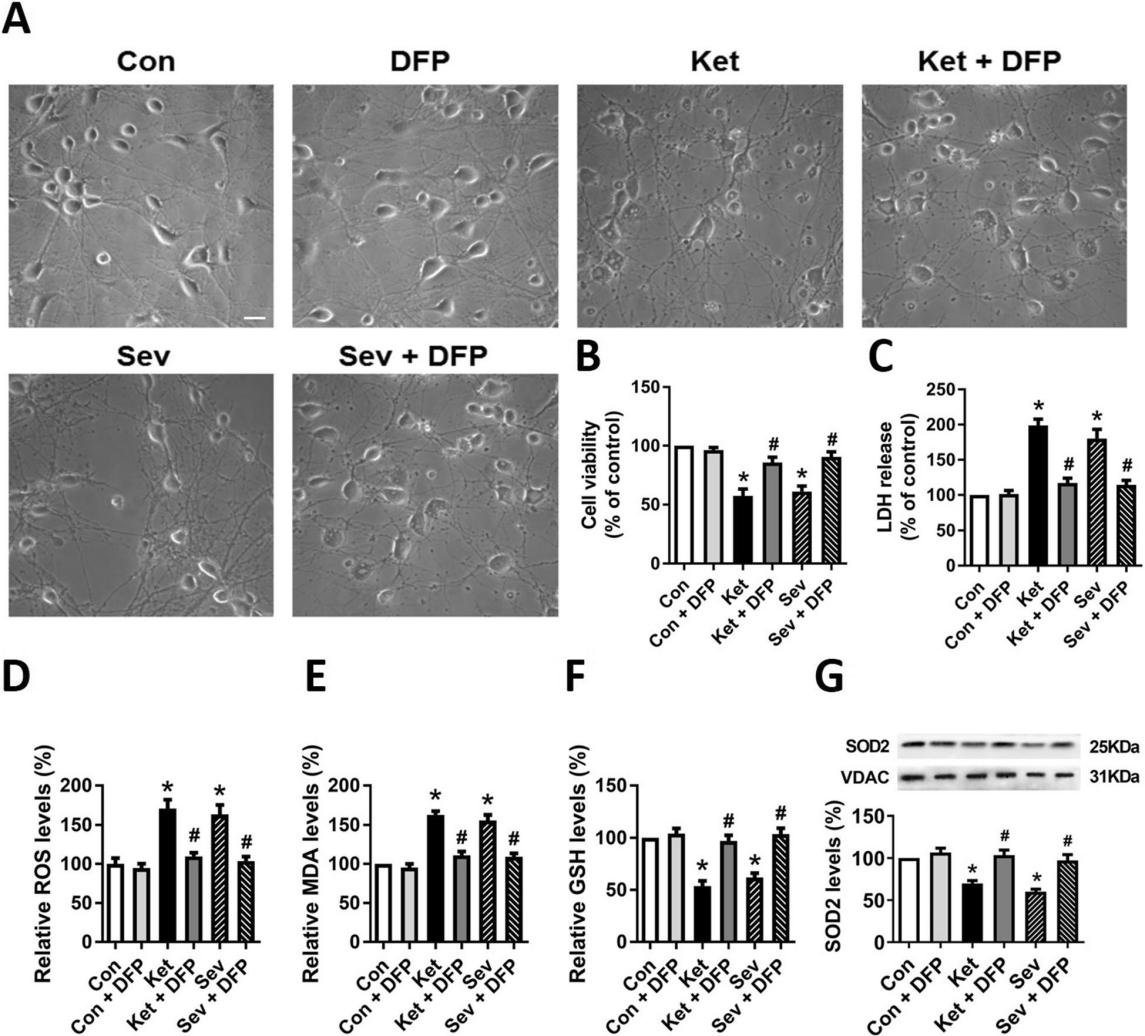
GA exposure induces the phenotypes of iron-mediated ferroptosis. **a** Neuronal morphology. The morphological changes of rat hippocampal neurons were observed under the microscopy at × 200 magnification. Scale bar 25 μm. **b** Cell viability and **c** lactate dehydrogenase (LDH) release by measurement of LDH activity in the culture medium, representing cytotoxicity from the GA-induced cellular damage 6 h after different treatments. **d**–**g** Ferroptosis-related biomarkers: ROS, MDA, GSH, and SOD. Data are presented as mean ± SEM (*n* = 6); *p < 0.05 compared with Con group; ^#^*p* < 0.05 compared with the GA (Ket or Sev) group

Although the characteristics of ferroptosis have not been perfectly understood, the key biomarkers include iron dependence and the stress levels of lipid peroxidation [32]. Here, we showed that GA treatment induced cells to exhibit condensed and ruptured mitochondria (Fig. 3d), which also belongs to the morphological characteristics of ferroptotic cells [33]. Not only the cell morphology (Fig. 4a), viability (Fig. 4b), and cytotoxicity (Fig. 4c) but also other biomarkers (Fig. 4d–g) all matched the phenotypes which represents the process of ferroptosis, and they were all rescued by iron chelation. For instance, the general reactive oxygen species (ROS, Fig. 4d), the final lipid peroxidation product MDA (Fig. 4e), reduced GSH (Fig. 4f) content, and even the scavenger SOD2 (Fig. 4g) altered after GA exposure and reversed after DFP treatment. These results support the notion that ferroptosis is derived from iron-dependent neurotoxicity after GA exposure.

### Chelating neurotoxic iron ameliorates GA-induced cognitive deficits

**Fig. 5:**
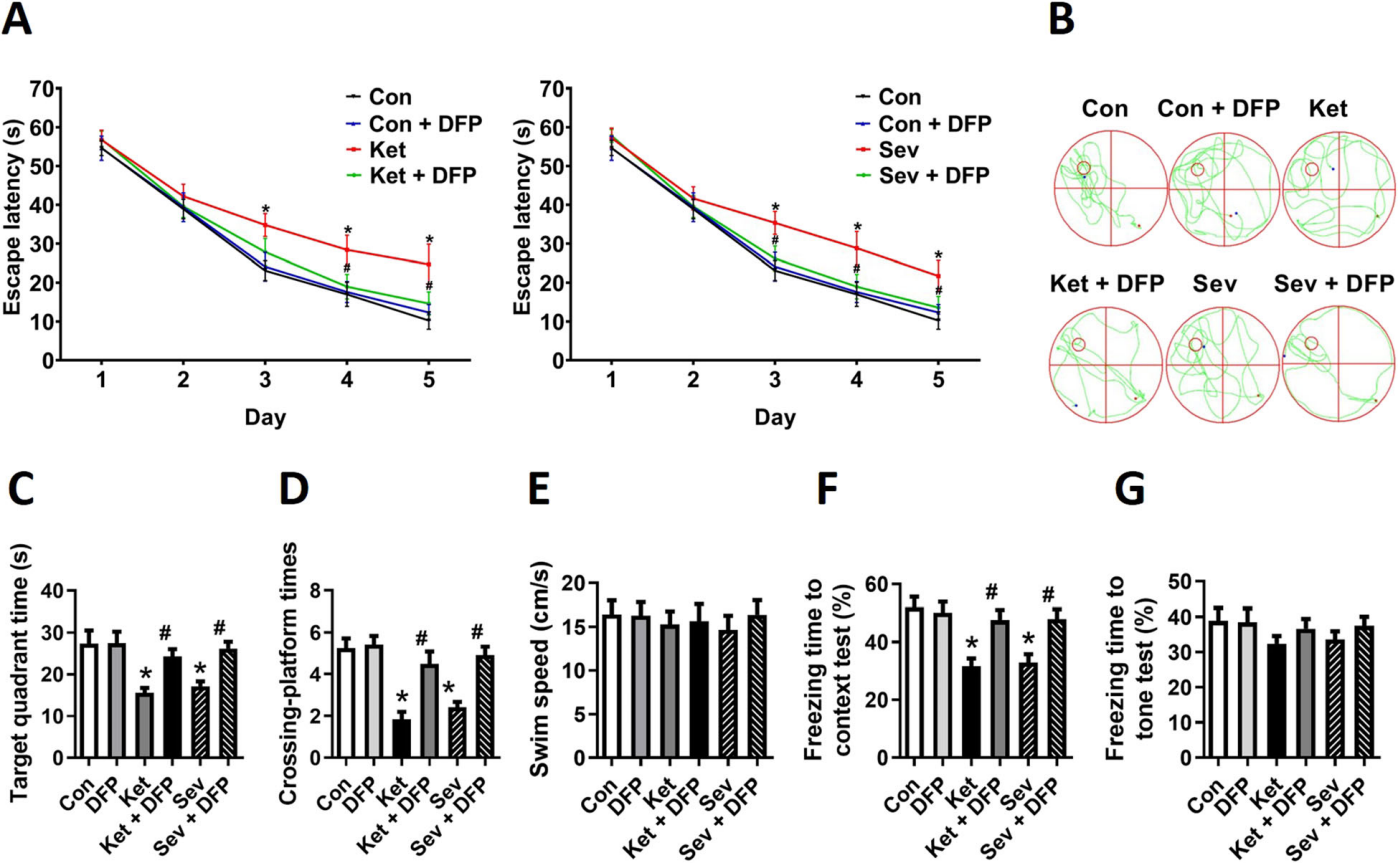
Restriction of neurotoxic iron accumulation ameliorates GA-induced cognitive deficits in the adolescent rats and aged mice. **a** Escape latency during the spatial training of MWM for 5 consecutive days. Rat pups received different treatments including GA or/and DFP exposure daily for three consecutive days from PND 6 to PND 8. The MWM tests were performed at PND 60. **b** Representative swimming trajectory of the rats. **c** Time spent in the target quadrant, **d** crossing platform times, and **e** swim speed in the probe trial of MWM. **f** Freezing time to context and **g** freezing time to tone in the fear conditioning tests. The fear conditioning tests for the aged mice were performed 24 h after GA exposure. **a**–**e** for adolescent rats; **f** and **g** for aged mice. Data are presented as mean ± SEM (*n* = 12 animals/group); *p < 0.05 compared with Con group; ^#^*p* < 0.05 compared with the GA (Ket or Sev) group

To estimate the extent to which GA-induced cognitive outcomes were attributed to iron overload, the adolescent rats were engaged in MWM tests on the indicated days after GA exposure with DFP pretreatment. The MWM tests showed long escape latency in the spatial training tests (Fig. 5a) and decreased target quadrant time and crossing platform times (Fig. 5b–d) in probe trial in adolescent rats after exposure to either ketamine or sevoflurane. Interestingly, the swim speed was not found significantly changed (Fig. 5e). For aged mice, fear conditioning tests were performed and showed, comparing to the control, a decreased percentage of freezing time in the 24-h context test (Fig. 5f, g), which reflects the impairment of hippocampus-dependent memory, after ketamine or sevoflurane treatment. Beyond our expectation, DFP pretreatment remarkably improved the performance in the behavioural tests (Fig. 5). These results indicate that chelating neurotoxic iron with DFP ameliorated GA-induced cognitive deficits and suggest that iron restriction might provide a preventative effect for paediatric patients undertaking GA and might have a therapeutic effect for POCD treatment.

### NMDAR-mediated iron transport pathway is involved in GA-induced iron overload

**Fig. 6:**
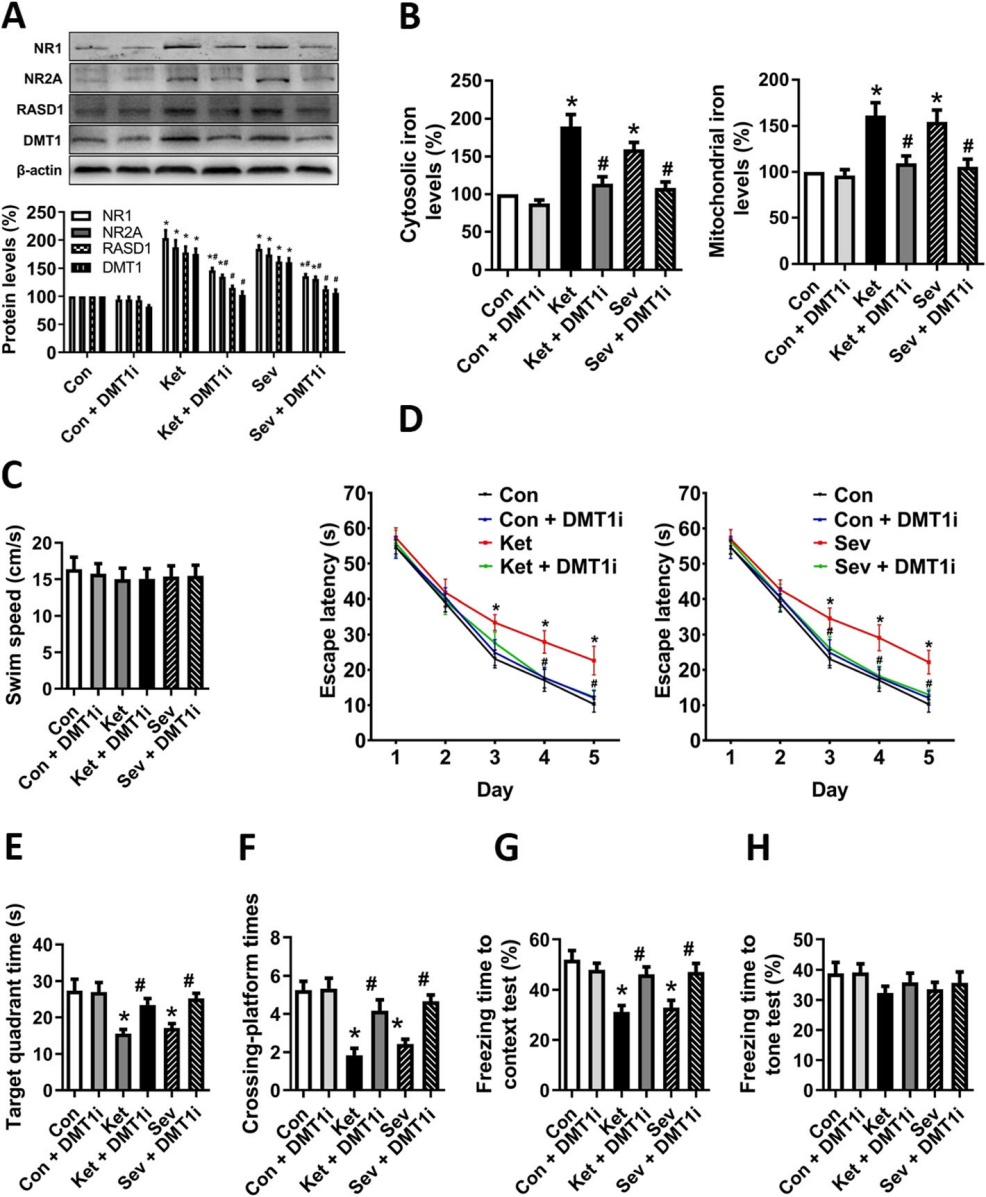
GA-induced activation of NMDAR-RASD1-mediated DMT1 iron uptake signalling pathway. **a** To inhibit NMDAR-RASD1-mediated DMT1 iron uptake signalling pathway, DMT1i was added to the culture medium 30 min before GA exposure. Protein levels of NMDAR, RASD1, and DMT1 were determined by Western blot. A representative image set is presented. **b** The cytosolic and mitochondrial iron levels of hippocampal neurons. **c** Swim speed, **d** escape latency, **e** time spent in the target quadrant, and **f** crossing platform times in MWM tests. **g** Freezing time to context and **h** freezing time to tone in the fear conditioning tests. **a** and **b** for hippocampal neurons; **c**–**f** for adolescent rats; **g** and **h** for aged mice. Data are presented as mean ± SEM (*n* = 12 animals/group); *p < 0.05 compared with Con group; ^#^*p* < 0.05 compared with the GA (Ket or Sev) group

Based on the above results, we further explored the molecular mechanism underlying GA-mediated iron overload in the brain. NMDAR is one of the two main targets to the GAs. Previous experiments revealed that ketamine or sevoflurane treatment upregulates the expression of NMDAR subunits (compensatory regulation as a consequence of continued or prolonged NMDAR blockade) [34–36]. Moreover, NMDAR upregulation leads to the activation of RASD1 (also called as Dexras1), a novel GTPase, which interacts with iron importer DMT1 and enhances DMT1-mediated iron uptake and iron releasing from lysosome [8, 37]. Here, we found increased expression of NMDAR1 (NR1) and NMDAR2A (NR2A), as well as RASD1 and DMT1, after 6 h ketamine or sevoflurane treatment (Fig. 6a). To reveal whether GA-induced iron accumulation could be via the enhanced iron uptake, a DMT1-specific inhibitor (DMT1i) [19, 20] was utilized. Intriguingly, DMT1i pretreatment before ketamine or sevoflurane exposure significantly suppressed the expression of all the tested components, NR1, NR2, RASD1, and even DMT1 (Fig. 6a), and iron contents in cytosol and mitochondria were also maintained evenly (Fig. 6b) in rat hippocampal neuronal culture. Amazingly, without any swim speed change (Fig. 6c), DMT1i ameliorated the GA-induced cognitive impairments in vivo in all the tested assays including escape latency (Fig. 6d), target quadrant time (Fig. 6e) and crossing platform times (Fig. 6f) in MWM tests of the rats, and freezing time (Fig. 6f) in fear conditioning test of the aged mice. The freezing time to tone test (Fig. 6h) did not show the difference after DMT1i treatment. Our data, overall, confirmed the effect of GA-induced iron neurotoxicity and demonstrated that blockade of iron uptake would provide a feedback effect on NMDAR-mediated signalling to protect neurons from damage.

## Discussion

This study provides the first integrative evidence that intravenous and inhalational GAs, such as ketamine and sevoflurane, may induce iron overload in the hippocampus of developing and aged brains to be involved in the cognitive deficits, likely, through neuronal ferroptosis. Our study shed new light on understanding the mechanism of GAs to generate neurotoxicity, at least partly, through modulation of iron metabolism in the brain.

The developing or aged brain has several significant differences from the adult brain that provide a physiological basis for enhanced resistance to insults, including exposure to perioperative stressors [4, 5]. Recent studies have demonstrated that excess iron may be detrimental for neural development, behavioural and cognitive functioning, particularly, at the extremes of age [10, 13, 14]. In this study, we found that both ketamine and sevoflurane disrupted iron homeostasis by affecting the expression of proteins involved in iron uptake further to induce cytosolic and mitochondrial iron accumulation in the cultured hippocampal neurons and in the hippocampus of developing rats and aged mice. Interestingly, pretreatment with DFP successfully ameliorated the turbulence of iron metabolism and attenuated the ketamine- or sevoflurane-induced neuronal toxicity. Previous studies have revealed that surgical trauma induces iron accumulation in the hippocampus and that iron chelator DFO, whose mode of action is still under investigation, protects against neuroinflammation and cognitive impairments in an aged rodent model of POCD [38, 39]. However, the blood-brain barrier is thought to be relatively impermeable to DFO [40]. Unlike DFO, DFP, a small positively charged molecule (only 139 Da), is neutral in the circulation and, whether free or bound to iron, readily penetrates cells [41]. Therefore, we used DFP to chelate the accumulated cellular iron in brain or ex vivo neurons, which is in line with the clinical first-line treatment for removing and/or preventing iron accumulation in the brain [42].

Mitochondria are important organelles in all cell types, particularly in the neurons. Mitochondrial dysfunction has been previously shown to link to the earliest pathogenesis of GA-induced cognitive impairments in developing or aged mammalian brain [17, 29]. On the one hand, we confirmed our previous findings including the mitochondrial dysfunction after GA exposure [43]; on the other hand, we observed that pretreatment with DFP prior to GA exposure significantly attenuated GA-induced effects on mitochondrial function and dynamics. This means that GA-induced impairment of mitochondria is directly associated with iron accumulation, which is consistent with the previous studies in other iron-overload models [44]. Therefore, we can draw a rough conclusion that iron toxicity is involved in the mechanism underlying GA-induced mitochondrial dysfunction.

Ferroptosis is a relatively new form of regulated cell death. Though still more to be unfolded, it involves iron dependence and lethal lipid-based ROS generation. Emerging data suggest that ferroptosis is one, if not the main, driver of cell death in cancer and neurodegenerative diseases like Parkinson’s disease, Alzheimer’s disease, and Friedreich’s ataxia [15, 45]. In the present study, our results showed that ketamine or sevoflurane initiated neuronal death, presenting the combination of a few ferroptotic biomarkers including iron dependence, increased lipid peroxidation, and decreased GSH content [32]. Among them, it is very attractive that iron restriction by iron chelation with DFP or blockage of iron uptake with DMT1i not only abolishes GA-induced ferroptosis but also resists GA-induced neurotoxicity and cognitive deficits. Both ways to limit the iron availability are to restrict NTBI in a short time frame, suggesting that the iron dependence of ferroptosis is rather NTBI- than other sources of iron dependence. Recently, it has been demonstrated that increase of DMT1 expression, not increased TfR1 or decreased Fpn1, may be mainly responsible for aging or disease-dependent increase of iron in brain [46], which may share the same reason as GA-induced iron overload in this study. The previous studies have demonstrated that anaesthesia has a significant impact on phosphorylation of tau and on neuropathology of cognitive decline in Alzheimer’s disease [47]. A recent study has addressed that iron deposition leads to hyperphosphorylation of tau and impairs cognition in mic e[48]. Thus, hyperphosphorylation of tau, as a result of iron overload, is supposed to be another mechanism of GA-induced cognitive deficits.

Evidence proved that NMDAR upregulation could promote iron overload and iron-induced neurotoxicity by enhancing RASD1-DMT1-mediated iron uptake [8] and iron releasing from the lysosome [9]. In the present study, we provided direct evidence that ketamine or sevoflurane exposure caused an upregulation of NMDAR subunits, which might be a compensatory upregulation of NMDAR subunits [34–36]. Accompanying with that, both ketamine and sevoflurane induced upregulation of RASD1, which could form a ternary complex with PAP7 and DMT1 and thus enhance the ability of NMDAR to activate DMT1 for iron uptake [8, 37]. The activation was inhibited by administration of DMT1 inhibitor DMT1i in this study to prevent iron accumulation in neurons, which attenuated iron-induced neurotoxicity. Interestingly, DMT1i treatment reduced the protein levels of NR1, NR2A, RASD1, and DMT1. One possible mechanism is that DMT1i ameliorates GA-induced iron overload and consequently provides a feedback to counteract the GA-induced compensatory upregulation of NMDAR. At the end, it causes the downregulation of NR1, NR2A, and RASD1. The similar feedback regulation was also observed previously [9]. Therefore, it is possible that modulation of iron level is an effective strategy for prevention from NMDAR-induced neurotoxicity.

## Conclusion

This is the first time, at least to our knowledge, to reveal that iron overload is involved in GA-induced neurotoxicity and cognitive deficits. This work suggests a new strategy that local depletion of iron or restriction of iron influx might be beneficial for the treatment of GA or iron overload-induced neurodevelopmental or neurodegenerative toxicity.

## Data Availability

All data generated in this study are included in this manuscript.

## Abbreviations

GA: General anaesthesia
GAs: General anaesthetics
POCD: Postoperative cognitive dysfunction
NMDA: *N*-Methyl-d-aspartate
GABA: Gamma-aminobutyric acid
DMT1: Divalent metal transporter 1
NTBI: Non-transferrin-bound iron
DFP: Deferiprone
IRP: Iron regulatory proteins
TfR1: Transferrin receptors 1
Mfrn: Mitoferrin
mPTP: Mitochondrial permeability transition pore
MMP: Mitochondrial membrane potential
